# Utility of near-infrared fluorescent clip for the robot-assisted gastrectomy: Report of 2 cases (case series)

**DOI:** 10.1016/j.ijscr.2024.110576

**Published:** 2024-11-12

**Authors:** Kenji Takeshita, Naoto Takahashi, Yuta Takano, Naoki Toya, Fumiaki Yano, Ken Eto

**Affiliations:** a Department of Surgery, The Jikei University Kashiwa Hospital, Japan; b Department of Surgery, The Jikei University, Tokyo, Japan

**Keywords:** Near-infrared fluorescent (NIRF) clip, Gastric cancer, Robot-assisted gastrectomy, Case series

## Abstract

**Introduction and importance:**

The importance of preoperative tumor site marking has increased over the years, as the method of intraoperative primary lesion identification and determination of resection margins is one factor determining whether oncological safety and function-preserving gastrectomy are possible during surgery. We hypothesize that preoperative placement of the near-infrared fluorescent (NIRF) clip, ZEOCLIP FS, near the oral incision line of the gastric tumor will allow for Firefly recognition of the NIRF clip on da Vinci during surgery and easy determination of the tumor location and incision line. Hence, we report on two cases in which the procedure was performed.

**Case presentation:**

Case 1: A 62-year-old woman was diagnosed with early gastric cancer of 35 mm in size located in the greater curvature of the gastric angle and underwent robot-assisted distal gastrectomy. NIRF clips were placed around the negative biopsy-confirmed area on the tumor's oral side by endoscopy on the day before surgery. The clips were identified intraoperatively in Firefly mode, and we performed gastrectomy without using an intraoperative endoscope. Case 2: A 60-year-old man was diagnosed with early gastric cancer 40 mm in size on the anterior wall of the gastric angle and underwent robot-assisted distal gastrectomy. Similarly, NIRF clips were placed around the site of negative biopsy confirmation the day before surgery. NIRF clips were identified, and we performed gastrectomy.

**Clinical discussion:**

The time taken to mark the gastric resection line after activating the Firefly imaging system was 120 and 154 s, respectively, and intraoperative endoscopy was not required. The advantage of our two-step method is that a surgeon can mark the clips the day before the surgery, even if they are not endoscopists. Increasing the recognition rate of fluorescent clips and preventing their remains are future issues.

**Conclusion:**

Based on the results of the above two cases, ZEOCLIP FS is influential in determining the tumor's location and the resection line.

## Highlights

• Identifying the tumor and resection line in robot-assisted gastrectomy is challenging, but ZWOCLIP FS® can mark the resection line in minutes.

## 1 Backgrounds

The importance of preoperative tumor site marking has increased over the years, especially in East Asia, where early gastric cancer accounts for a large proportion of cases, and the method of intraoperative primary lesion identification and determination of resection margins is an essential factor in determining whether oncological safety and function-preserving gastrectomy are possible during surgery [1]. Therefore, marking clip methods have attracted attention recently, especially the usefulness of fluorescent clips used in laparoscopic surgery [2,3]. However, they have the disadvantage of being difficult to visualize in robotic surgery.

ZEOCLIP FS® (Zeon Medical, Tokyo, Japan), a near-infrared fluorescent clip, can be positioned as a marker by grasping tissue with a clip, and the position of the clip can be recognized by fluorescence observation using near-infrared light as excitation light. The da Vinci Xi (Intuitive Surgical, California, USA) is equipped with “Firefly,” a near-infrared fluorescent laparoscopic system that uses an 805 nm laser as excitation light. However, the conventional ZEOCLIP does not sufficiently support this excitation light. Therefore, a new near-infrared fluorescent clip was developed in December 2021 to comply with Firefly by adjusting the fluorescent dye concentration (boron-dipyrromethene) in ZEOCLIP FS [4].

We report two cases of robot-assisted distal gastrectomy using ZEOCLIP FS. We perform preoperative marking clips in two steps because ZEOCLIP FS has a 24-hour limit for grasping force and fluorescence. First, mark a 20 mm oral side location from the endoscopically diagnosable tumor margin with two or three hemostatic clips. At that time, a negative biopsy will be performed to ensure that there is no cancer invasion in the planned excision line. Finally, the day before surgery, place the ZEOCLIP FS on the anterior and posterior wall near the marking clip.

## 2 Presentation of cases

**Fig. 1:**
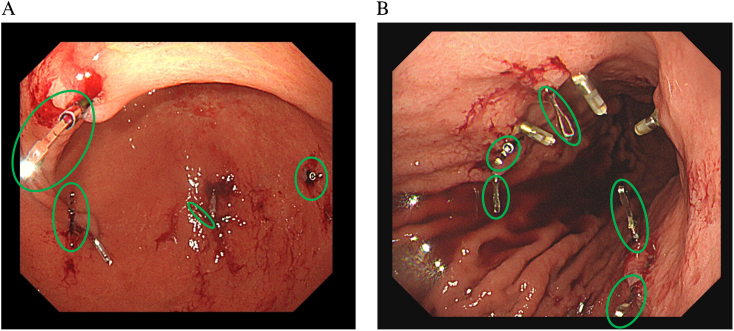
A: Four ZEOCLIP FS were placed in the anterior and posterior walls around the tumor (green circles). B: Three ZEOCLIP FS were placed around the negative confirmed point on the anterior wall and two on the posterior wall (green circles). (For interpretation of the references to colour in this figure legend, the reader is referred to the web version of this article.)

**Fig. 2:**
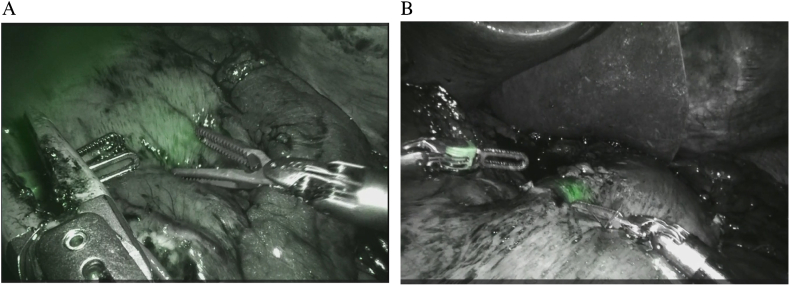
A: NIRF clips placed in the anterior and posterior walls of the stomach were identified intraoperatively in Firefly mode. B: One NIRF clip placed in the anterior wall of the stomach was identified intraoperatively in Firefly mode.

Case 1: A 62-year-old woman was diagnosed with early gastric cancer (sig > por, cT1a, N0, M0), 35 mm in size, located in the greater curvature of the gastric angle, and underwent robot-assisted distal gastrectomy. Four ZEOCLIP FS were placed in the anterior and posterior walls (Fig. 1-A). NIRF clips were confirmed intraoperatively in Firefly mode and resected without an intraoperative endoscopy (Fig. 2-A). It took to mark the gastric resection line after activating the Firefly imaging system was 120 s. Operation time was 531 min, and blood loss was 10 ml. No intraoperative complications occurred.

**Fig. 3:**
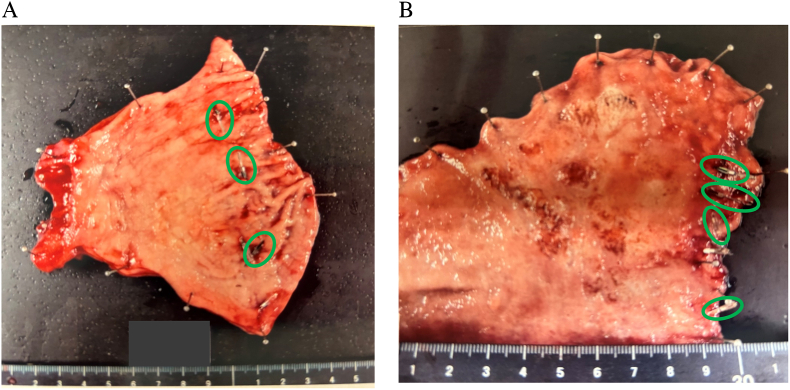
A: Resection specimen with marking clips (Case 1). Endoscopic diagnosis showed it to be 35 mm large, but pathology showed 25 × 25 mm (Green circles: ZEOCLIP FS). B: Resection specimen with marking clips (Case 2). Endoscopic diagnosis showed a tumor 40 mm in size, but pathology showed a 65 × 51 mm tumor with type 4-like spread (Green circles: ZEOCLIP FS). (For interpretation of the references to colour in this figure legend, the reader is referred to the web version of this article.)

No adverse events were related to the use of NIRF clips. The proximal margin was 35 mm (Fig. 3-A). The postoperative course was fine, and the patient was discharged on postoperative day 17.

Case 2: A 60-year-old man was diagnosed with 40 mm size early gastric cancer (tub1 > tub2, cT1b, N0, M0) in the anterior wall of the gastric angle. Five ZEOCLIP FS were placed around the confirmed point on the anterior and posterior wall the day before surgery (Fig. 1-B). The clip was confirmed intraoperatively in Firefly mode and resected without an intraoperative endoscopy (Fig. 2-B). It took to mark the gastric resection line after activating the Firefly imaging system was 154 s. However, one NIRF clip remained in the remnant stomach. No additional resection was performed because all original hemostatic marking clips were on the resected side. Operation time was 515 min, and blood loss was 20 ml. There were no intraoperative complications. His postoperative course was fine, and he was discharged from the hospital on postoperative day 12. The final pathological examination showed that the proximal margin was 10 mm (Fig. 3-B). The clip in the remaining stomach was retrieved by endoscopy intraoperatively. This case series has been reported in line with the PROCESS Guideline [5].

## 3 Discussion

Today, fully laparoscopic and robot-assisted gastrectomy are widely used. However, identifying the primary tumor and determining the gastrectomy line remains difficult, and various techniques are used [[6], [7], [8], [9], [10], [11], [12], [13], [14], [15]]. Current laparoscopic techniques include intraoperative endoscopic confirmation, Indian ink injection [6,7], and intraoperative C-arm fluoroscopy [7] to confirm clip location. Intraoperative endoscopy is still widely used but requires a skilled endoscopist to use the endoscope during surgery. Insertion of the endoscope in the supine position is usually more complicated, and the problem is that it is difficult to perform the procedure simultaneously with laparoscopy in the pneumoperitoneal state.

Indian ink Injections may stain the lesion darkly, interfering with naked-eye observation [8]. C-arm fluoroscopy or portable X-ray equipment [[9], [10], [11]] can objectively determine the resection line. However, they have the disadvantages of increased operative time for equipment preparation and the need for radiation-protective clothing. Subsequently, indocyanine green (ICG)-based marking has been used in some centers [12,13]. Under visible light, the fluorescent region of ICG does not interfere with naked-eye observation because of its different wavelengths. Still, pharmaceutical restrictions on the endoscopic injection of ICG and protocols differ among institutions regarding ICG concentration and other factors and must be standardized [14]. Also, as with India ink, the fluorescence of ICG may spread too widely from the injection point [15], but NIRF clips do not need to consider the spread of ICG, allowing for more pinpoint marking.

In the two cases presented here, robot-assisted distal resection was performed using the XEOCLIP FS without an intraoperative endoscope. The operative times for the two surgeries were 531 and 515 min, longer than the standard operating time. This is because the da Vinci operation was introduced at the initial stage (within 10 cases), and the surgeon was still immature in his surgical operations [16]. However, marking the gastric resection line after activating the Firefly imaging system was 120 and 154 s, respectively. Thus, the excision line could be determined quickly, a significant advantage of not requiring an endoscope during the procedure.

The first case was completed without any adverse events related to fluorescent clips. However, in Case 2, one fluorescent clip remained in the residual stomach, and only one could be seen intraoperatively. ZEOCLIP FS tends to have a relatively low recognition rate in the stomach wall. Also, in 2023, a report showed that in RDG surgery with NIRF-clip marking for gastric cancer using the da Vinci built-in Firefly imaging system, the clip recognition rate for gastric cancer cases was 75 % in 12 of 16 cases [17]. As reported, the stomach wall is thicker than the colon wall, and the thickness of the digestive tract wall that can be recognized using excitation light is limited to 10 mm, which may make recognition difficult in Firefly mode, depending on the viewing angle [18]. Therefore, it is necessary to extend the gastric wall with forceps and make the camera angle close to the vertical to the gastric wall. Reports of laparoscopic surgery using fluorescent clips showed that stretching the gastric wall by pumping air into the nasogastric tube increases recognition rates [19,20]. During endoscopic clipping, the fluorescent resin portion of the clip should be as close as possible to the serosal surface, allowing a deeper bite into the mucosal surface. On the other hand, we implanted four or five NIRF clips to improve the visibility of the fluorescent clips. As a result, at least one clip could be identified, and the excision line could be quickly determined. There are several problems with using so many fluorescent clips. The first is that it is expensive at 12,000 yen for a clip. Currently, the Japanese national health insurance system does not cover fluorescent clips, placing a significant financial burden on hospitals. While advanced medical equipment often incurs high initial installation costs, the demand for such technology is rising. In an aging society, there is a growing need for more precise and effective treatment methods. Consequently, the market for products like fluorescent clips is expanding, with hopes that technological innovation will lead to the development of similar, low-cost alternatives. Second, the more clips they have, the more likely they will be left behind. We believe placing two clips close to each other on the front wall and a third on the posterior wall to avoid clip residual clips is appropriate.

The advantage of our two-step method, despite the burden of multiple endoscopies, is that a surgeon, even if they are not an endoscopist, can mark the clips the day before the surgery as long as they know where the fluorescent clip should be placed. Takahashi reported an average of 1.76 days for the recognition group and 2.57 days for the non-recognition group regarding the number of days between clip application and surgery. The recognition rate decreased as the time interval between clip application and surgery got longer [17]. Fluorescent clips with an extended fluorescence efficacy period are also expected to be developed to reduce the number of endoscopic examinations.

These two cases suggest that in robotic surgery using Firefly, as in conventional laparoscopic surgery, marking with NIRF clip using ZEOCLIP FS is influential in determining the tumor location and line of resection and contributes to shortening the operative time because an intraoperative endoscope is not needed.

## Abbreviations

NIRF: near-infrared fluorescent
ICG: Indocyanine Green

## Declaration of Generative AI and AI-assisted technologies in the writing process

We do not use generative AI and AI-assisted technologies in the writing process.

## Consent for publication

The patient has given consent for the publication of images. Written informed consent was obtained from the patients for publication and any accompanying images. A copy of written consent is available for review by the Editor-in-Chief of this journal on request.

## Ethics approval and consent to participate

This study followed the principles of the Declaration of Helsinki. In my institution (The Jikei University Ethics Committee), no ethical committee clearance or approval was needed to write a case report.

## Funding

We received no support from any funding.

## Authors' contributions

NT, KT, and YT were responsible for the patient's clinical management and data acquisition. KT and NT were responsible for drafting the manuscript and interpreting the data. NT, FY, and KE were accountable for the critical revision of the manuscript. All authors read and approved the final manuscript.

## Declaration of competing interest

The authors declare that they have no competing interests.

## Data Availability

All data generated or analyzed during this study are included in this published article.
